# The Network for Analysing Longitudinal Population-based HIV/AIDS data on Africa (ALPHA): Data on mortality, by HIV status and stage on the HIV care continuum, among the general population in seven longitudinal studies between 1989 and 2014

**DOI:** 10.12688/gatesopenres.12753.1

**Published:** 2017-11-06

**Authors:** Emma Slaymaker, Estelle McLean, Alison Wringe, Clara Calvert, Milly Marston, Georges Reniers, Chodziwadziwa Whiteson Kabudula, Amelia Crampin, Alison Price, Denna Michael, Mark Urassa, Daniel Kwaro, Maquins Sewe, Jeffrey W. Eaton, Rebecca Rhead, Jessica Nakiyingi-Miiro, Tom Lutalo, Dorean Nabukalu, Kobus Herbst, Victoria Hosegood, Basia Zaba

**Affiliations:** 1 Department of Population Health, London School of Hygiene & Tropical Medicine, London, WC1E 7HT, UK; 2 Department of Infectious Disease Epidemiology, London School of Hygiene & Tropical Medicine, London, WC1E 7HT, UK; 3 Malawi Epidemiology and Intervention Research Unit, Lilongwe, Malawi; 4 School of Public Health, University of the Witwatersrand, Johannesburg, 2000, South Africa; 5 MRC/Wits Rural Public Health and Health Transitions Research Unit (Agincourt), School of Public Health, Faculty of Health Sciences, University of the Witwatersrand, Johannesburg, 2000, South Africa; 6 National Institute for Medical Research, Mwanza, Tanzania; 7 Kenya Medical Research Institute, Kisumu, Kenya; 8 Department of Infectious Disease Epidemiology, Imperial College London, London, W2 1PG, UK; 9 MRC/UVRI Uganda Research Unit on AIDS, Entebbe, Uganda; 10 Rakai Health Sciences Program, Entebbe, Uganda; 11 Africa Health Research Institute, Durban, 4001, South Africa; 12 Department of Social Statistics & Demography, University of Southampton, Southhampton, SO17 1BJ, UK

**Keywords:** HIV, Sub-Saharan Africa, HIV incidence, Mortality, HIV care continuum, Anti-retroviral therapy, Longitudinal, Population based

## Abstract

Timely progression of people living with HIV (PLHIV) from the point of infection through the pathway from diagnosis to treatment is important in ensuring effective care and treatment of HIV and preventing HIV-related deaths and onwards transmission of infection.  Reliable, population-based estimates of new infections are difficult to obtain for the generalised epidemics in sub-Saharan Africa.  Mortality data indicate disease burden and, if disaggregated along the continuum from diagnosis to treatment, can also reflect the coverage and quality of different HIV services.  Neither routine statistics nor observational clinical studies can estimate mortality prior to linkage to care nor following disengagement from care.  For this, population-based data are required.

The Network for Analysing Longitudinal Population-based HIV/AIDS data on Africa brings together studies in Kenya, Malawi, South Africa, Tanzania, Uganda, and Zimbabwe.  Eight studies have the necessary data to estimate mortality by HIV status, and seven can estimate mortality at different stages of the HIV care continuum.  This data note describes a harmonised dataset containing anonymised individual-level information on survival by HIV status for adults aged 15 and above. Among PLHIV, the dataset provides information on survival during different periods: prior to diagnosis of infection; following diagnosis but before linkage to care; in pre-antiretroviral treatment (ART) care; in the first six months after ART initiation; among people continuously on ART for 6+ months; and among people who have ever interrupted ART.

## Introduction

In 2014, an estimated 25. 8 million people in sub-Saharan Africa were living with HIV (PLHIV), representing 70% of the global total, and 790,000 HIV-related deaths occurred ^1^. A third of these deaths occurred in six countries: Kenya, Malawi, Tanzania, South Africa, Uganda and Zimbabwe ^2^. The network for Analysing Longitudinal Population-based data on HIV/AIDS in Africa ( ALPHA) ^3^ brings together health and demographic surveillance systems (HDSS) in these six sub-Saharan African countries, which have longitudinal data on participants and their HIV status.

These studies combine demographic, survey, clinical and verbal autopsy data to provide comprehensive, population-based estimates of the incidence of new HIV infections, the number of PLHIV, their experience of diagnosis and treatment, and their survival. For many PLHIV, these data permit estimation of seroconversion date and dating of diagnosis of infection, initiation of antiretroviral therapy (ART) and interruption of treatment.

Since the rollout of ART, overall adult mortality ^2, 4– 10^, and that among PLHIV both on and off treatment, has fallen substantially ^11^. However, the continuing high HIV mortality burden indicates that improvements in HIV testing, care and treatment are still needed.

For PLHIV, the continuum of care describes the steps necessary to achieve viral suppression ^2, 12^. Key transition points include diagnosis, assessment of ART eligibility, initiation of ART and viral suppression. Failure to progress in a timely manner from infection to diagnosis and from diagnosis to treatment will have a deleterious impact on mortality rates and onward HIV transmission.

Tracking PLHIV along each step of the HIV care continuum is challenging for several reasons ^2^: Data on ART initiation and adherence are commonly available and readily collated from ART clinics with computerised records, but data on time of diagnosis and confirmation of eligibility for ART are less frequently recorded in formats that permit aggregation of those data for monitoring purposes. Furthermore, dates of seroconversion are not routinely estimated in the course of HIV testing or clinical care.

This data note describes a dataset containing harmonised survival data for DSS participants aged 15 years and above from eight studies in six countries over the period 1989–2014. The person-time of PLHIV is categorised by stage on the HIV care continuum: a) had undiagnosed infection, b) had learned their HIV status, but had yet to enrol in care, c) had received some pre-ART care, but had not (yet) started treatment, d) were receiving ART continuously, and e) had experienced an interruption in ART. Using these data, it is possible to estimate mortality at different stages of the HIV care continuum, and progression along the continuum.

## Methods

### Participating studies

Eight ALPHA Network studies could estimate mortality by HIV status over periods ranging from 4 to 26 years, and seven had data on HIV diagnosis and treatment: Karonga (Malawi) ^13^; Kisesa (Tanzania) ^14^; Kisumu (Kenya) ^15^; Manicaland (Zimbabwe) ^16^; Masaka (Uganda) ^17^; Rakai (Uganda) ^18^; uMkhanyakude (South Africa) ^19^. Agincourt in South Africa ^20^ had mortality data and partial HIV status data. The period with the most comparable data is 2005–14.

These studies are mainly ongoing rural HDSS ^21^ and collect demographic data on the entire population resident in specific geographical areas via frequent, focussed enquiries, and conduct regular home- or community-based HIV testing of the resident adult population. Additional socio-economic, HIV-related and sexual behaviour data are collected via questionnaire-based surveys. In uMkhanyakude there were no self-reported data on uptake of HIV care and treatment.

All resident adults are eligible for HIV testing, with the exception of Kisumu and Manicaland where HIV testing is restricted to a smaller, representative, cohort within the study. For the period in question, only Karonga and Kisumu returned HIV results automatically to participants, but all participants had the opportunity to find out their status by opting to receive the results of the research test or by undergoing HIV testing and counselling (HTC) in parallel with the research testing and in accordance with contemporary local standards for HTC, described elsewhere ^3^.

The ALPHA network partners are independent research institutions, and began fieldwork at different times and with differing research priorities. Consequently, there is variation in study protocols and the data available. Recently, these studies have begun to link their population-based data with clinical data from local health facilities, but the information available from health facility records and the methods used to link these data to the HDSS vary. Four studies (Kisesa, Masaka, Rakai and uMkhanyakude) linked visit-level data from local ART clinics to the HDSS data. Administrative changes in clinics interrupted data linkage with the HDSS after 2012 in Rakai and 2013 in uMkhanyakude. Two studies (Karonga and Kisumu) had summary information from ART clinics linked to HDSS records. No clinical data were available for Manicaland, but some information on treatment was available from self-reported survey data. Detailed information is given in Box 1.

**Box 1.** Details of clinic data available from each site and the methods used to link these data with the population-based data.

**Karonga** Links between the health and demographic surveillance system (HDSS) and clinical records were made by study fieldworkers in all 16 clinics that provide ART in the area. Those attending were interviewed and identified in the HDSS database. Initially, links were made only in ART clinics, so there is little information on pre-ART care. Clinical records were summarised every 6 months, and data to 2016 were shared with ALPHA. **Kisesa** Links between the HDSS and clinical records were made in two ways. Probabilistic links based on names, date of birth and place of residence were made for records from two HIV clinics from mid-2015 onwards. Individuals identified as HIV positive in the sero-survey or at routine HIV testing and counselling (HTC) in the HDSSstudy area were referred to local care providers, and their referral was tracked to enable ongoing linkage between clinical records and the HDSS. **Kisumu** A summary of patients’ records was made annually since 2009 from the 19 clinics providing HIV care. Probabilistic and deterministic links were made using the Kenyan national identity number and other identifying information. **Masaka** Since 2012, all HIV positive participants have been routinely referred to the clinic run by the study which uses the same identifieras the research project. Pre-2012, those in selected clinic studies and those attending HTC were referred to the clinic and linked. **Rakai** Between 2004 and 2013, Rakai had good coverage of 19 HIV care clinics in the area covered by the HDSS and used probabilistic methods to link data with the HDSS. However, in 2013 the system for sharing clinic data with the HDSS changed and data are not available from that point onwards. **uMkhanyakude** Deterministic links between the HDSS and 21 HIV care and treatment clinics serving the HDSS area were made using South African national identity numbers and referrals tracking from the HDSS. Probabilistic record linkage was done whenever national ID numbers were not available.

It is worth noting that the period covered by these data includes the early phase of ART provision in these communities, and the decentralisation of treatment. Provision of treatment went ahead of strengthening clinic systems for monitoring and reporting on ART initiation, adherence and other relevant clinical measures. Many of the clinics that provide treatment to HDSS participants do not have electronic medical records (EMR) not least due to a lack of reliable electricity. During this period, most clinics could not easily undertake CD4 counts, and there was no viral load testing or resistance screening. Thus the standard of care for a typical clinic visit may compare unfavourably to that of high-income settings at the time. In addition, the lack of EMR means that some observations were not systematically recorded, for example, in many cases consultations were summarised in freehand notes. Such information is cumbersome and expensive to extract from handwritten logbooks and registers, and has thus not been included.

The information needed to identify time spent in pre-ART care (still an important stage on the continuum during the period covered by these data) was not available for PLHIV in Manicaland (via self-report) or for most PLHIV in Karonga. Additionally, nobody in Manicaland is classed as having interrupted ART because the survey question on interruption of treatment did not permit dating of this event.

Local researchers supplied the dates at which ART first became available in at least one government health facility in each HDSS area, and the date when the ART rollout to all local health facilities designated as ART providers under national ART policy guidelines was complete.

### Transitions and stages on the continuum of care for HIV

The primary purpose for constructing this dataset was to describe and compare mortality at different stages of the care continuum over time and between studies. Given the data available across the different studies, we defined stages of the HIV care continuum based on: The estimated date of seroconversion from HIV negative to positive and the date of diagnosis of HIV infection, enrolment in HIV care, initiation of ART, stabilisation on ART and first interruption of ART. High quality data to describe temporary or permanent disengagement from pre-ART care or date of first ART eligibility were not available. The stages of the HIV care continuum are the intervals between these transitions. The care continuum, as described above, is a one-way system since people cannot return to a category after leaving it. Those who had a gap in treatment of longer than one month and those who had discontinued ART, at any time after initiation, were allocated to a group of people who had experienced an interruption in treatment, which included those who subsequently resumed ART.

Viral load was not routinely measured in the clinics serving these populations and so we could not identify people who achieved viral suppression. In lieu, we used a period of time on ART (more than six months without interruption), and used that classification as a proxy for stabilisation on treatment.

### Data sources and availability

Data from each study were harmonised to standard specifications for analysis ( Table 1, also see http://alpha.lshtm.ac.uk/metadata/ for the detailed specifications) although not all datasets were available from every study.

**Table 1.** ALPHA standard datasets available from each site.

	ALPHA standard datasets:	Karonga	Kisesa	Kisumu	Manicaland	Masaka	Rakai	uMkhanyakude
1	Information on residence in the study area, including dates of birth, migration and death.	●	●	●	●	●	●	●
2	History of HIV testing, including dates of tests, circumstance in which test was carried out, final test result, and whether or not the test result was returned to the participant.	●	●	●	●	●	●	●
3	Verbal autopsy data.	●	●	●	●	●	●	●
4	Self-reported information, from periodic surveys, on use of HIV testing services, disclosure of HIV status, use of HIV care and treatment services, ART use and interruption of ART.	●	●	●	●	●	●	
5	HIV clinic records on enrolment in care and ART history.	●	●	●		●	●	●

### Analytical methods for preparation of the dataset

Data from all HDSS participants, regardless of HIV status, were prepared for survival analysis based on residency data using Stata 15 ^22^. Entry into the study was on the 15 ^th^ birthday or the date of first in-migration to the HDSS area (if already aged over 15), or the date of enumeration in the baseline study round. Exit was the date of out-migration or death. Gaps in residency due to temporary migration were recorded as such in the analysis. Surviving residents were censored at the time of their last HDSS observation. The dataset is organised to have multiple records for each individual, with each record corresponding to a short period of exposure which permits estimation of mortality by age, calendar time and HIV, diagnosis and treatment status. In Stata ^22^ terminology, this means the data have been stset and stsplit (see page 377 of the Survival Analysis Reference Manual ^23^).

### Assignment of HIV status

Person-time was allocated to one of three HIV status categories (unknown, negative, positive). Individuals could move through all three categories during study follow-up. HIV status information was available from a) periodic research HIV tests, b) linked clinic data, and c) self-report. Prior to first HIV test, individual HIV status was classed as unknown. The time between two negative tests was classed as negative and for the purpose of calculating mortality among HIV-negative adults, the period after the last HIV negative test was classed as negative for a short period, after which person-time was classed as unknown. For each study, the period of time classed as negative after the last negative test was based on the observed local incidence rates and the cut-off point was defined as the time by which 5% of people of the same age and sex had seroconverted. This ranged from 2 to 50 years in the East African studies (mean 10·8) and from 1 to 50 years in the Southern African studies (mean 2·8). Individuals were assigned as HIV positive immediately after the first positive research test. People of hitherto unknown HIV status who were identified in HIV or ART clinics were classified as positive from the date of the first visit to the clinic. In Kisumu, participants could report their HIV status in lieu of a test and those results were used for people without a test result (less than 3% of participants).

People who were observed to seroconvert were treated exactly the same way as all other people who had tested negative and were categorised as positive only after the first positive test. Those for whom the interval between the last negative and first positive tests exceeded the cut-off were classified as unknown for a period prior to the first positive test.

### Assignment of person time and deaths to stages on the HIV care continuum

Information on HIV diagnosis status, use of HIV-related care and ART history came from:

a) receipt of HIV test result following periodic HDSS research tests
b) self-report of HIV test history, diagnosis, pre-ART care and ART use, including dates
c) linked clinic data, primarily on pre-ART care and ART

The information used from each source is detailed in Table 2. A greater amount of information on experience of HTC, and thus diagnosis date, was available from self-reported and study data than from clinic data, whereas ART histories were derived almost entirely from clinic data.

**Table 2.** Categories on the HIV care and treatment continuum.

Person years assigned as:	Description		Information used	
		Study research tests	Self-report	Clinic data
Undiagnosed	Never been told has HIV	Never given test results by study since first testing positive	Never received a test result * Not received a test result since first recorded positive test	Not used, cannot confirm undiagnosed status
Diagnosed	Has received >=1 positive test result	Has been given a positive test result by study	Reports having received a test result after date of first recorded positive test	Not used
In care	Has attended (>=1 occasion) an HIV service or care clinic but has never had ART	-	Reports having attended HIV care services at least once. No restriction on type of service or frequency of visit. (ART naïve)	Attended an HIV care clinic on at least one occasion (ART naïve)
Early ART	Started ART within the past 6 months, no breaks	-	First 6 months after reported start date for ART and no gaps reported/ before first gap	First six months of continuous attendance at ART clinic
Stable ART	Been on ART longer than 6 months without a break	-	More than 6 months after reported start date for ART and no gaps reported (includes time before first gap)	After six months of continuous attendance at ART clinic *or, if* *initiation not captured,* continuous attendance since first visit in someone who initiated ART elsewhere or before the clinic’s data were linked with the HDSS
Interrupted ART	Started ART but has experienced one or more gaps of 1 month or more in treatment	-	After first reported interruption in ART	
Unknown	Insufficient information to allocate to any of the above groups	-		

* Distinction between taking an HIV test and receiving the results is not possible in some rounds in Masaka.

Some individuals had information on a transition from more than one source, but no source of data is clearly superior. Clinic data did not contain information on diagnosis and did not include people who used clinics outside the study area. Self-reported data may quickly become outdated, but can include interactions with a wide range of service providers if PLHIV are willing to disclose their status to the interviewer. We considered using algorithms to give more weight to data from either clinic data or self-reported data for the transitions that could be identified in both data sources, but concluded that it was not possible to systematically deem one source to be more reliable than the other in all circumstances. Therefore, we took the earliest reported instance of any of the transitions as the date for that change regardless of the source of that data. We discarded self-reported dates that were implausible (such as, ART initiation before birth), but imposed no other constraints. Thus if, for example, a self-reported start date for ART was before the first positive test in the study, we backdated the HIV positive person time to the reported date of ART initiation.

Some participants retrospectively reported the dates when they made a transition (first positive diagnosis, linked to care, started ART, interrupted ART). For this analysis of mortality we did not use the retrospectively reported date but instead used the date of first report. This was to avoid biasing the data towards survivors, who were able to make a retrospective report of their progression along the continuum which those who had died before the survey could not do.

HIV positive person-time was allocated to one of the continuum stages listed in Table 2 based on a synthesis of all the information available for each person and each transition. If at least one source indicated that an individual had made a transition, then their person-time following that transition was allocated to the subsequent stage on the continuum. This process was repeated if information was available on subsequent transitions. After the last observed transition, all the remaining person-time was allocated to the last recorded stage. Transitions that occurred when the individual was not under observation in the study (due to temporary out migration) were recorded as occurring at the time that the individual returned to observation to avoid biasing the data towards temporary, rather than permanent, outmigrants and to survivors.

In addition to information on the dates when individuals made the various transitions, we also had dates at which a particular transition had not already been made from null reports in self-reported survey data (i.e. does not know HIV status, has never been to HIV care, has never taken ART, has never stopped taking ART) and from clinic history (has never started ART, has never stopped taking ART). Therefore person-time allocated to each individual’s final stage can be disaggregated into time when the individual is known not to have made the transition to the next stage and the person-time contributed by people for whom there is no evidence regarding the next transition. For mortality analyses, we did not separate the person-time in the final stage into these two groups, or use this information to censor the follow-up time, because there is a zero probability of mortality in the time before the final null report.

### Continuity of care and adherence to ART

We identified anyone who ever self-reported or had been observed to have a break in treatment and distinguished these individuals from those who never interrupted ART. We were not able to make this same distinction for pre-ART care due to a lack of data for PLHIV in this stage. Based on clinic data, continuous attendance was defined as PLHIV attending appointments not more than one month after: a) next recorded scheduled visit, b) time when their pills ran out (based on records of pills dispensed at previous visit), or c) the interval between visits specified in national guidelines. This interval ranged from 1 to 3 months. PLHIV for whom we had only self-reported data regarding ART were assumed to be continuously on ART until they reported stopping or interrupting ART. When this was reported, or when a gap was identified in clinic data, PLHIV were moved to the last stage: interrupted ART.

### Relative size and annual coverage of the different studies

For each study site, the total number of individuals included in this dataset is determined by the size of the population participating in demographic surveillance and the length of time the study has been in existence. The number of PLHIV included is further determined by the HIV prevalence and participation in survey rounds that included HIV testing. The proportion of participants who were HIV positive at the most recent survey ranged from 6% in Karonga to 33% in uMkhanyakude.

**Figure 1.**
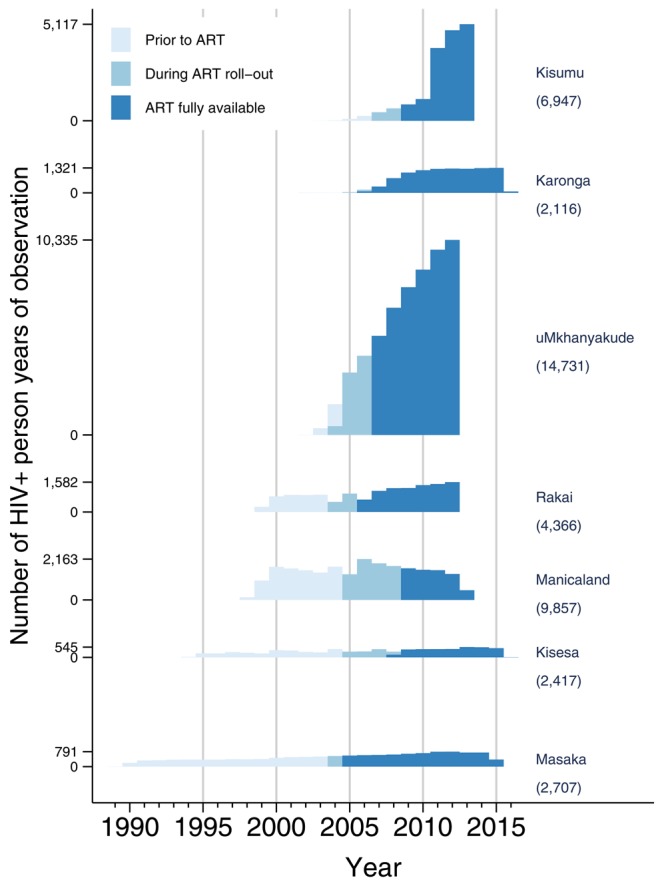
HIV positive person time by site and calendar year. Numbers in brackets give total number of PLHIV ever identified.

The average number of PLHIV under observation in each HDSS, for the periods i) before the introduction of ART; ii) during ART roll-out; and iii) once ART was widely available, are shown in Figure 1.

In terms of the number of individuals followed, the largest is uMkhanyakude in South Africa (approximately 63,000 adults in early 2012), followed by Kisumu in Kenya (circa 47,000 adults in early 2012), which began population-wide HIV testing in 2005 and 2008, respectively. We used only part of the Kisumu data, that from Gem district, which has more HIV data than the other two districts. The studies with the longest follow up are Masaka (beginning 1989) in Uganda and Kisesa in Tanzania (beginning 1994), which had, respectively, approximately 10,000 and 16,000 adults under observation at the start of 2012.

The introduction of ART occurred at different times in the different sites and the interval between introduction and widespread availability varied markedly ranging from one year in Masaka to 3·5 years in Kisesa and Manicaland in Zimbabwe.

### Description of variables

The variables and coding, where relevant, are given in Supplementary File 1.

## Dataset validation

### Availability of linked clinic data

The ability to link clinical records to HDSS data varies between sites and has changed over time. Table 3 shows, by study, the number of PLHIV observed during 2005, 2010 and 2014 and the percentage for whom clinic data were available. The proportion of people with linked clinic data available represents the extent to which PLHIV are diagnosed and enrolled in care, and also the ability of the study site to link DSS and clinical records. In Kisesa, the total number of PLHIV fluctuates markedly because new HIV positives are identified once every 2–3 years, but exits due to death and out-migration are recorded approximately every 6 months in the HDSS.

**Table 3.** Number of people living with HIV (PLHIV) and the percentage linked to clinical records by study and year.

Study	2005		2010		2014	
	Number PLHIV	% linked to clinic	Number PLHIV	% linked to clinic	Number PLHIV	% linked to clinic
Karonga	124	67.7	1296	54.6	1339	70.1
Kisesa	395	2.5	584	13.9	570	8.4
Manicaland	2571	0.0	2222	0.0		
Masaka	622	54.5	772	60.1	769	61.0
Rakai	1149	33.6	1609	47.9	1135	44.7
uMkhanyakude	4034	5.5	9705	46.5	11584	48.4
Kisumu	183	100.0	1938	65.3		

### Data availability over time, by HDSS and transition along the continuum

**Figure 2.**
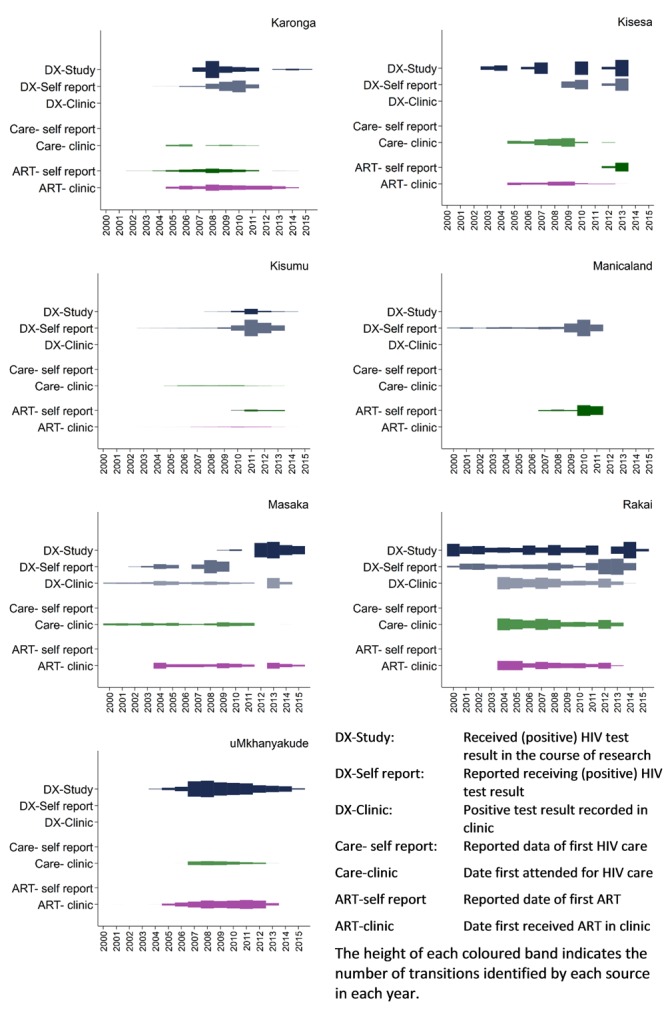
Data available from each study by data source and continuum stage, over time.

There were important differences between the studies in the availability of data. The time covered by each data source is shown in Figure 2 and is disaggregated for each transition. The absolute numbers of transitions observed within each study varies substantially and so, to facilitate cross-study comparison, the height of each coloured band indicates, within a study, the relative number of transitions in each year, for each source, expressed as a proportion of the largest group in the study (at any time point).

### Impact of intermittent reporting on data quality

The validity of our results is determined by the timeliness of our data, and the degree to which we base our classification of person-years on outdated information. ALPHA studies collect survey data every one to three years, depending on study site, and the time elapsed between individual reports may be longer because a) individuals do not always participate in every round, and b) questions may have been omitted from some rounds.

Person-time after an observed transition is, by default, allocated to the next stage on the continuum. For example, after a positive test where there is no record of results being reported to the individual, person-time is allocated to the undiagnosed stage until the date of diagnosis/linkage to care or ART, if this occurs and is recorded. We may have one (or more) reports that the person has not yet learned their HIV status and that allows us greater certainty about the allocation of time to the undiagnosed stage. Following such a report, the chance of misclassification (the person learning their status but we fail to capture that information) increases with time since the last report.

**Figure 3.**
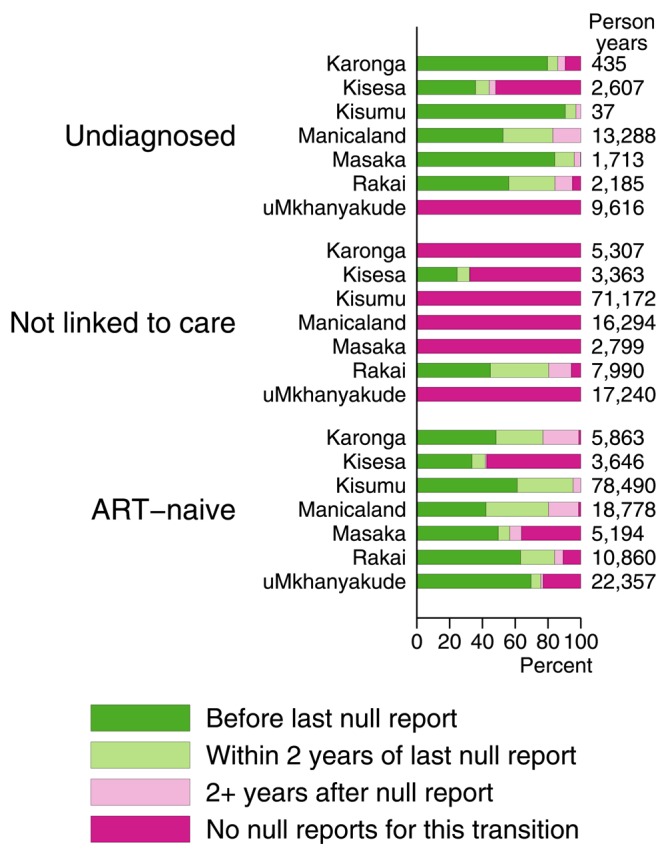
HIV positive person years prior to each transition along the continuum by the existence and recency of the information used to place them.

Examining the distribution of these null reports (a report stating that a transition has not been made) allows us to see how certain we are regarding allocation of person time to each category. Figure 3 shows the distribution of person-time amongst PLHIV that was allocated to stages on the HIV care continuum based on not having made certain transitions: not diagnosed, not linked to care and not on ART (ART naïve). The total person-time in these states is summed and the figure shows the proportions of that time that were a) before a null report regarding the subsequent transition, b) less than two years after the last null report regarding the subsequent transition, and c) more than two years after the last null report. The fourth category shows person-time contributed by individuals for whom we have never had any information regarding the subsequent transition.

Pre-diagnosis has the greatest share of person-time prior to the last report, the lowest proportion where there is no evidence either way, and a high proportion of person time within two years of the last report on diagnosis status. This is because in most studies this information is collected systematically at the same time as the HIV testing is conducted for research purposes and, in all studies except uMkhanyakude, participants are asked about testing history.

The transition to pre-ART care is the most poorly described, in part because pre-ART care was not as widely or systematically available as treatment. Five studies had no null information because this transition is only recorded by the arrival of the individual in an HIV care clinic. In Kisesa and Rakai this information is collected in self-report, which is why there is some person time known to be pre-transition.

The transition to ART is well described, with the exception of Kisesa, but despite that in most studies a substantial minority of person-years are classified as ART-naive by default because there is no evidence either way. Note that many of those individuals do have information on diagnosis.

## Ethical policies

Best practice regarding HIV testing for research purposes has changed during the lifetime of these studies. The earliest data collection made use of informed consent without disclosure. After treatment became available, research testing was accompanied by opportunities for participants to learn their status using a variety of locally approved protocols.

Local ethical approval for these analyses was obtained by each study. The harmonisation and analysis of the pooled mortality data was approved by the ethics committee of the London School of Hygiene and Tropical Medicine (ref 6467) on 23 ^rd^ August 2013.

## Consent

Written informed consent for analysis of the participants’ anonymised details and publication of the results was obtained from the participants or, for verbal autopsy data, from a relative of the deceased participant.

## Data availability

The ALPHA Network harmonises the data produced by a diverse group of longitudinal HIV studies. Ownership of the data rests with the studies, or in some cases with the national government. The member studies have shared their data with the Network, but have not transferred ownership. The dataset is maintained by Emma Slaymaker, but access to the data is conditional on obtaining permission to use each study’s data. Requests for permission to use the data should be directed to the ALPHA Network contact person in each study:

**Table:** 

Study	Contact	Point of contact
Karonga	Amelia (Mia) Crampin	http://meiru.lshtm.ac.uk/
Kisesa	Mark Urassa	via ALPHA
Kisumu	Daniel Kwaro	dkwaro@kemricdc.org
Manicaland	Simon Gregson	http://www. manicalandhivproject. org/data-access.html
Masaka		via ALPHA
Rakai	Tom Lutalo	tlutalo@rhsp.org
uMkhayakude	via the Africa Health Research Institute data repository	help@africacentre.ac.za

The ALPHA Network can facilitate such enquiries ( http://alpha.lshtm.ac.uk/). Requests should state the name of the dataset: ALPHA_Gates_ready_2015_pooled_2_Aug_2017.dta.

## Supplementary material

Supplementary File 1: Description of variables.
