# Supplementary material for: The Network for Analysing Longitudinal Population-based HIV/AIDS data on Africa (ALPHA): Data on mortality, by HIV status and stage on the HIV care continuum, among the general population in seven longitudinal studies between 1989 and 2014
Source: Gates Open Res. 2017 Nov 6;1:4. [Version 1] doi: 10.12688/gatesopenres.12753.1 (PMC5841576; doi:10.12688/gatesopenres.12753.1)
Supplement: Supplementary file 1 [file gatesopenres-1-13813-s0000.tgz › 528cea6f-56eb-4b8c-82a2-56bd4eb4bf3e.docx]

# Supplementary File 1: Description of variables

| **Variable name** | **Description** | **Coding** |
| --- | --- | --- |
| site | Site (alphabetical order) | \| 1 \| Agincourt \| \| --- \| --- \| \| 3 \| Karonga \| \| 4 \| Kisesa \| \| 5 \| Kisumu \| \| 6 \| Manicaland \| \| 7 \| Masaka \| \| 8 \| Rakai \| \| 9 \| uMkhanyakude \| |
| study_name | Site (ALPHA order) | \| 1 \| Karonga \| \| --- \| --- \| \| 2 \| Kisesa \| \| 3 \| Manicaland \| \| 4 \| Masaka \| \| 5 \| Rakai \| \| 6 \| uMkhanyakude \| \| 7 \| Agincourt \| \| 8 \| Kisumu \| |
| idno | Pooled ALPHA individual identifier |  |
| sex | Sex | \| 1 \| Men \| \| --- \| --- \| \| 2 \| Women \| |
| dob | Date of birth |  |
| region | Eastern or Southern African site | \| 1 \| Eastern \| \| --- \| --- \| \| 2 \| Southern \| |
| last_neg_date | Date of last negative HIV test |  |
| frst_pos_date | Date of first positive HIV test |  |
| exit | Date of exit from study |  |
| entry | Date of entry into study |  |
| failure | Whether died at the end of this time segment (binary) |  |
| _st | Stata system variable used for survival analysis |  |
| _t0 | Stata system variable: analysis time when record begins |  |
| _t | Stata system variable: analysis time when record ends |  |
| _d | Stata system variable:1 if failure; 0 if censored |  |
| years_one | Calendar year |  |
| fiveyear | 5-calendar year group | \| 1 \| 1989-1994 \| \| --- \| --- \| \| 2 \| 1995-1999 \| \| 3 \| 2000-2004 \| \| 4 \| 2005-2009 \| \| 5 \| 2010-2014 \| \| 6 \| 2015-2019 \| |
| age | Single year |  |
| agegrp | Five year age group | \| 1 \| 15-19 \| \| --- \| --- \| \| 2 \| 20-24 \| \| 3 \| 25-29 \| \| 4 \| 30-34 \| \| 5 \| 35-39 \| \| 6 \| 40-44 \| \| 7 \| 45-49 \| \| 8 \| 50-54 \| \| 9 \| 55-59 \| \| 10 \| 60+ \| |
| timepostneg | Negative test expiry date in years |  |
| hivstatus_detail | HIV status in this time segment | \| 1 \| Negative \| \| --- \| --- \| \| 2 \| Positive \| \| 3 \| Pre positive (1st test +ive) \| \| 4 \| Post Negative < years \| \| 5 \| Unknown, never tested \| \| 6 \| Before first negative test \| \| 7 \| Seroconv interval \| \| 8 \| Post negative after years \| |
| hivstatus_broad | HIV status in this time segment, grouped | \| 1 \| Negative \| \| --- \| --- \| \| 2 \| Positive \| \| 3 \| Unknown \| |
| allinfo_treat_pyramid | HIV, diagnosis and treatment status in this time segment | \| 0 \| No HIV status \| \| --- \| --- \| \| 1 \| HIV negative \| \| 2 \| HIV+ no further information \| \| 3 \| HIV+ not diagnosed \| \| 4 \| HIV+ diagnosed \| \| 5 \| HIV+ attended services \| \| 6 \| HIV+ on ART <6m \| \| 7 \| HIV+ on ART 6m+ \| \| 8 \| HIV+ on ART w gaps \| |
| hivtreat | Treatment status, distinguishes stable and interrupted ART | \| 0 \| No more info \| \| --- \| --- \| \| 1 \| HIV negative \| \| 2 \| Never treated \| \| 3 \| Early ART \| \| 4 \| Stable ART \| \| 5 \| Interrupted ART \| |
| hivevertreat | Treatment status, ART naive or exposed | \| 0 \| No more info \| \| --- \| --- \| \| 1 \| HIV negative \| \| 2 \| Never treated \| \| 3 \| Had ART \| |
| preg_at_art_start | Whether gave birth within 6 months of ART initiation. (binary) |  |
| hiv_update_flag | Whether HIV status from research tests has been updated based on clinic data | \| 0 \| No change from study test \| \| --- \| --- \| \| 1 \| Updated from clinic data \| \| 2 \| Updated from SR \| |
| art_available | ART available in study area (binary) |  |
| art_avail_cat | Availability of ART in the study area in this time segment | \| 0 \| None \| \| --- \| --- \| \| 1 \| Available \| \| 2 \| Fully available \| |
