# Supplementary material for: The Network for Analysing Longitudinal Population-based HIV/AIDS data on Africa (ALPHA): Data on mortality, by HIV status and stage on the HIV care continuum, among the general population in seven longitudinal studies between 1989 and 2014
Source: Gates Open Res. 2017 Nov 6;1:4. [Version 1] doi: 10.12688/gatesopenres.12753.1 (PMC5841576; doi:10.12688/gatesopenres.12753.1)
Supplement: Peer review history

# Peer review: Referee response for version 1

**DOI:** 10.21956/gatesopenres.13813.r26102

**Published:** 2017-12-07

**Authors:** Tracy R. Glass

**Affiliations:** 1 Department of Clinical Research Unit, Swiss Tropical and Public Health Institute, Basel, Switzerland

This paper describes the valiant efforts of many people to collect quality data for research purposes. From experience in just one of these countrie’s HDSS data collection, I know the challenges they have faced.  I think they did a good job attempting to give an accurate picture of the data they collected and its limitations both for use and for drawing valid and generalizable conclusions.

I have read this submission. I believe that I have an appropriate level of expertise to confirm that it is of an acceptable scientific standard.

# Peer review: Referee response for version 1

**DOI:** 10.21956/gatesopenres.13813.r26075

**Published:** 2017-12-04

**Authors:** Matthias Egger

**Affiliations:** 1 Institute of Social and Preventive Medicine, University of Bern, Bern, Switzerland

This is the first time I review a Data Note for Gates Open research. According to the instructions for authors, “Data Notes are brief descriptions of research datasets that include details of why and how the data were created; they do not include any analyses or conclusions”. In general I felt the data note on the network for Analysing Longitudinal Population-based HIV/AIDS data on Africa (ALPHA) is well written and I have approved it. The report could be a bit more detailed and clear in some places, and I have made suggestions for improvements below. These can all be dealt with within a minor revision.

  

1. The longitudinal studies participating in ALPHA were set up for different reasons and with different research priorities. It would be helpful to detail these reasons and priorities in greater detail. What were the primary research objectives of the different studies? What is the primary research objectives of ALPHA?
2. Clearly, the studies were not established to study the HIV cascade of care and data were obtained from different sources, including documented test results from HDSS surveys, linked clinic data, and self-reported data. I was surprised to read that “it was not possible to systematically deem one source to be more reliable than the other …”. Isn’t a documented HIV test more reliable than a self-reported HIV status? Could this be clarified?
3. Following from above, if data from different sources were available, could the validity of the different sources be assessed, for example by considering documented test results as the gold standard. This might allow corrections, for example for social desirability bias. Please comment.
4. It would be useful to know how many participants had a negative HIV test before their positive test? Also, how many individuals were considered HIV positive because they attended HIV clinics, without any testing data? Can this be gleaned from Figure 2? Not entirely clear to me.
5. A time window of 1 month seems rather short to define interruption of treatment, and prone to misclassification due to data errors. Any single undocumented visit or pharmacy claim is likely to lead to misclassification of patients as interrupted treatment. Please comment on the choice of this window – usually longer windows are chosen (3 months or 6 months).
6. How exactly were the HDSSs and clinic data linked? I would have thought a data note would exactly be the place where some technical details should be provided on the type of probabilistic or deterministic linkage used.
7. The section on the Assignment of HIV status is not entirely clear. First the authors say that the period after the last HIV negative test was classed as negative for a short period (how short?) but then go on to state that the period of time classed as negative after the last negative test was based on the observed local incidence rates, that the cut-off point was defined as the time by which 5% of people had seroconverted (how was this threshold chosen?) and that this ranged from 2 to 50 years in the East African studies. Please clarify.
8. It would be interesting to see a discussion of how a cascade of care can be constructed using these data, including the definition of numerators and denominators at each step of the cascade, and what particular issues arise in the different studies.
9. Important limitations of the dataset include the lack of CD4 cell and viral load data, and the time period covered, which for some studies ends in 2012. This could be discussed in greater detail, particularly in the light of the recent changes in WHO guidelines (“treat all”).
10. Figure 2 needs more explanation and a scale for the horizontal bars that vary in width over time.
11. No datasets are provided and I could therefore not assess the question: “Are the datasets clearly presented in a useable and accessible format?”

I have read this submission. I believe that I have an appropriate level of expertise to confirm that it is of an acceptable scientific standard.

# Peer review: Referee response for version 1

**DOI:** 10.21956/gatesopenres.13813.r26079

**Published:** 2017-12-01

**Authors:** Mary-Ann Davies

**Affiliations:** 1 Centre for Infectious Disease Epidemiology and Research, University of Cape Town, Cape Town, South Africa

This is a well written article describing the data available in the ALPHA network that could potentially be used to assess mortality along the HIV care continuum.

 My specific comments are outlined below.

*Abstract:* *Among PLHIV, the dataset provides information on survival during different periods: prior to diagnosis of infection; following diagnosis but before linkage to care; in pre-antiretroviral treatment (ART) care; in the first six months after ART initiation; among people continuously on ART for 6+ months; and among people who have ever interrupted ART.*

 Given the sentence above – as well as the title of the article, I was expecting that the article would actually describe the survival (or its inverse – the mortality) during the different steps along the care continuum. However the article’s focus is actually on the data that is available to describe this mortality, rather than on survival or mortality itself. I suggest that the title and abstract are reworded to make this clear that the article is about the availability and quality of data from the alpha network that could be used to examine mortality along the HIV care continuum, rather than suggesting that the article will describe mortality itself. Perhaps the title should read: “The Network for Analysing Longitudinal Population-based HIV/AIDS data on Africa (ALPHA): Assessing the availability and quality of data on mortality…”

*Methods: Some participants retrospectively reported the dates when they made a transition (first positive diagnosis, linked to care, started ART, interrupted ART). For this analysis of mortality we did not use the retrospectively reported date but instead used the date of first report. This was to avoid biasing the data towards survivors, who were able to make a retrospective report of their progression along the continuum which those who had died before the survey could not do.*

 This is not completely clear. I fully understand the need to make some adjustment to avoid biasing the data towards survivors, but it is not clear to me how using the data of first report rather than the retrospectively reported date would achieve this. Surely this would mean that for many patients who retrospectively report they have been diagnosed, linked to care and started ART and interrupted ART it would appear that all of these events happened on the same day. Since this is an article about data validation it may also be helpful to actually show what the difference would be using the date of first report vs the retrospectively reported date.

*Dataset validation: Availability of linked clinical data.*

*The proportion of people with linked clinic data available represents the extent to which PLHIV are diagnosed and enrolled in care, and also the ability of the study site to link DSS and clinical records.*

 Since proportion of people linked to clinic is a combined outcome both of actually linking to care (a key clinical and programmatic outcome that we really want to measure accurately) and ability of the study site to link DSS and clinical records –which is a research function and has nothing to do with program performance or clinical outcomes, it seems odd to present this is a combined outcome which is really not meaningful, particularly when the percent linked to clinic ranges from 0-100%. I am not sure of the value of this table or of presenting the information unless it is possible to separate the two outcomes or to validate in some way for each site among the diagnosed people actually linked, what proportion the study is able to identify as linked.

 I found Figure 2 very difficult to interpret and am not sure what the key message is. I suggest this is made clearer. I think a table with absolute numbers may be clearer as well as some summary text highlighting the key messages.

 The article doesn’t seem to have a “Conclusion” or “Take home message”. This would be very helpful and I would suggest that the authors add this.

I have read this submission. I believe that I have an appropriate level of expertise to confirm that it is of an acceptable scientific standard, however I have significant reservations, as outlined above.
